# Supplementary material for: Are carbon emissions trading and green financial instruments synergistic? -Comprehensive quantitative research based on content analysis
Source: PLoS One. 2024 Mar 7;19(3):e0298601. doi: 10.1371/journal.pone.0298601 (PMC10919601; doi:10.1371/journal.pone.0298601)
Supplement: S1 Data — (ZIP) [file pone.0298601.s001.zip › Data/地级市Z_F工作报告.zip!/╔╜╬≈/╜·│╟╩╨/└┤╫╘╣1⁄2╓┌║┼í╛┬φ┐╦╩2╛▌═°í┐_2006.docx]

各位代表：

　　本届政府任期即将结束。现在，我代表市人民政府，向大会报告过去五年的工作，并对今年的工作提出建议，对《晋城市国民经济和社会发展第十一个五年规划纲要（草案）》作简要说明，请予审议，并请市政协委员和其他列席人员提出意见。

　　一、过去五年政府工作的回顾

　　过去的五年，既是实施“十五”计划的五年，也是本届政府任期的五年。五年来，市政府在市委的正确领导下，以邓小平理论和“三个代表”重要思想为指导，牢固树立和落实科学发展观，团结带领全市人民，开拓进取，争先发展，圆满完成了“十五”计划确定的主要目标，使全市经济社会发展迈上了新的台阶。

　　──城市综合竞争力显著增强。全市生产总值由2000年的146亿元增加到2005年的316亿元，年均递增12.6%；财政总收入由2000年的11.6亿元增加到2005年的61.7亿元，年均递增39.8%。其中一般预算收入由2000年的4.5亿元增加到2005年的20.1亿元，年均递增34.7%；全社会固定资产投资五年累计达到444亿元，年均递增17.4%；“十五”末金融机构各项存款余额达到504亿元，其中城乡居民储蓄存款余额达到290亿元。同时，我们先后摘取了全国卫生先进市、全国科技进步先进市、国家园林城市、中国优秀旅游城市、全国文明城市创建工作先进市、全国社会治安综合治理优秀市和山西省环保模范市等多项桂冠，有效地提升了我市的知名度和影响力，成为展示晋城形象、扩大晋城影响的重要名片。可以说，过去的五年，既是我市经济建设扎实推进、综合实力有效提升的五年，也是我市获得全国全省性荣誉数量最多、分量最重的五年。

　　──经济结构调整顺利推进。五年来，按照“一产调优，二产调强，三产调大”的思路，努力推进经济结构调整。农业结构日趋合理。在稳定粮食综合生产能力的同时，着力壮大畜牧、蚕桑、蔬菜、小杂粮、干鲜果、中药材等区域特色农业。尤其是畜牧业发展迅速，2005年肉、蛋、奶产量分别是2000年的1. 9倍、1.7倍和3. 6倍。农业产业化步伐加快，有6家农业产业化龙头企业进入全省“百龙企业”行列。工业整体素质明显增强。煤炭产业通过“关小改中建大”，生产集中度和技术装备水平有效提高。“十五”期间，共关闭有证但不达规模的小煤矿209个，关闭无证私开矿36个。到“十五”末，全市累计建成年产90万吨以上矿井9个，60万吨矿井8个，30万吨矿井48个，骨干矿井煤炭产量占到全市总产量的50%以上，煤炭资源回采率、入洗率和加工转化率分别达到45%、25％和20％；电力产业发展加快。阳电一期工程建成投产，二期工程开工建设，5个国家级小水电工程进展顺利，国投华实晋城热电联供项目和鲁能电厂项目正在积极争取国家的核准。五年新增发电装机容量221万千瓦，累计达到240万千瓦，实现了由输煤变输电的重大跨越。电力已成为我市仅次于煤炭的第二大产业；煤化工产业以大颗粒尿素为依托，积极延伸产业链条，迈出了新的步伐。晋城煤化工、天脊晋城化工、兰花科创、晋丰化工、天脊中化等大颗粒尿素项目和丹峰化工甲醇项目建成投产，晋煤集团和中联公司煤层气项目投入商业运行。2005年底，全市已形成230万吨大颗粒尿素和25万吨甲醇生产能力；冶铸产业升级转化步伐加快，一批大容积高炉建成投产，开发区国际铸造园区和高平泫氏、泽州金工、泽州晨辉等一批中高档铸造企业迅速崛起。福盛钢厂一、二期工程全面投产，生产能力达到100万吨；富士康晋城科技工业园建设进展顺利,标志着我市的先进制造业和电子信息业开始起步，对提升我市产业层次、优化产业结构具有重要意义；新型建材业稳步发展，阳城建瓷园区入区企业达到6 家，生产能力达到2100万平方米。第三产业继续壮大。特别是旅游产业以中国优秀旅游城市创建活动为载体，坚持整合旅游资源、配套硬件设施、改善服务环境和加大促销力度“四轮驱动”，皇城相府、王莽岭、珏山、山里泉等一批旅游景区迅速崛起，旅游资源优势正在转化为产业优势和经济优势。

　　──经济增长方式转变力度加大。五年来，以环境保护、生态建设和资源节约为重点，积极推进经济增长方式转变，取得了阶段性成果。深入开展环保专项治理，解决了一些危害群众健康的环境问题。取缔、改造市区燃煤锅炉148台，治理机动车尾气超标排放4900多辆，改装煤层气汽车600多辆，市区空气质量二级以上天数逐年递增，2005年实现天数为2000年的5.9倍。取缔、关闭工艺落后、污染严重的小冶炼、小建材、小化工等“土小”企业800多家，对27家重点企业实行了在线监测。五年累计完成造林85.9万亩，其中退耕还林51.6万亩，森林覆盖率由26.7%提高到33.6%，年均增长1.4个百分点。严厉打击私挖滥采，积极整合煤炭资源，促进了煤炭等矿产资源的合理开发和有效利用。落实最严格的耕地保护制度，保持了耕地总量的动态平衡。

　　──基础设施建设成效明显。五年来，一批关系增强经济发展后劲的重点工程相继竣工。晋焦高速公路、晋长高速公路、晋陵一级公路和端润一级公路建成通车，晋济高速公路开工建设，村村通水泥路或油路的目标基本实现，新增公路通车路程1505公里，其中高速公路128公里，公路交通主骨架基本形成。新建35千伏以上变电站20座，35千伏以上变电容量和输变电线路分别达到3892兆瓦和2208公里，比2000年末增长102.5%和40.4%。丹河源头引水工程基本完工，张峰水库、东焦河水库和磨滩水库建设进展顺利，五年累计新增供水能力2900万方，新增有效灌溉面积15万亩，解决和改善了农村50万人口的吃水困难。全市84个乡镇全部开通了光缆电视信号，95％的村实现了有线电视联网。

　　──城镇建设迈上新的台阶。中心城市建成区面积由2000年的23平方公里扩展到30平方公里，新建城市道路16条15.1公里。供水、供气、供热、供电和通讯能力有效提升，博物馆、体育场、游泳馆、文体宫相继建成，城市功能进一步完善。污水处理厂建成使用，垃圾处理厂开工建设，黄华街西区改造和“两河”治理工程基本完工。2005年建成区绿化覆盖率、绿地率和人均公共绿地面积分别达到39.6%、37%和12.5平方米，空气质量二级以上天数达到258天，人居环境明显改善。与此同时， 4个县城和嘉丰、巴公、北留等一批重点小城镇加快发展，基础设施和公共服务设施进一步完善。

　　──改革开放步伐加快。农村改革扎实推进，撤并乡镇41个，税费改革取得实质性突破，农业税及其附加全部免除，农民负担明显减轻。企业改革进一步深化。出台了深化企业改革的意见和相关配套文件，太印、气门破产工作全部结束，晋钢破产工作正在推进，煤炭资源整合和有偿使用工作有序开展。大公司、大集团战略有了质的突破，兰花科创进入“2005年度最具价值上市公司”50强，太行无烟煤集团挂牌运行。民营经济持续发展，民营经济增加值占全市生产总值的比重达到33.2%。国有资产管理体制改革迈出实质性步伐，新的国有资产监管机构组建完成。部门预算、国库集中支付、政府采购和收支两条线等财政体制改革取得显著成效。行政审批制度改革以及投融资、住房、社保等各项改革稳步推进。对外开放工作取得长足发展。积极实施“走出去、引进来”战略，多次组团参加厦洽会、乌洽会、高交会等一些重大的推介、招商和贸易活动，加强了与国内外的经济技术合作与交流。开发区建设步伐加快，特色工业园区从无到有，成为新的经济增长亮点。富士康集团、鲁能集团、天脊集团、中化集团等一批大企业、大集团先后落户我市，为经济发展注入了新的活力。积极实施“南下战略”，主动接轨中原城市群，融入中原经济圈，与美国工业重镇达拉斯市达成了建立友好城市意向，在加强与国内外的经济技术合作交流方面迈出了新的步伐。五年来，全市实际直接利用外资累计达到8275万美元，是“九五”期间的3.4倍；外贸进出口总额由2000年的1191万美元增加到2005年的10281万美元，年均递增53.9％。特别是去年一年增加进出口额5000多万美元，增幅位居全省第一。

　　──社会事业全面发展。五年来，累计组织实施各级各类科技项目549项，一大批先进适用技术得到推广，科技进步对经济社会发展的贡献率进一步提高。教育投入不断加大，改造中小学危房40.6万平方米，新建、改建和扩建农村标准寄宿制学校300所。教育资源配置逐步优化，基础教育、职业教育和成人教育得到加强，小学、初中入学率达到100%，高等教育从无到有，晋城职业技术学院形成一定规模。率先在全省免除了农村和城市低保家庭义务教育阶段学生的杂费。出台了《关于促进民办教育发展的若干意见》，推动了民办教育的发展。人才强市战略深入实施，人才使用机制不断完善。积极发展文化事业和文化产业，一批文化精品荣获全国、全省大奖，赵树理文学馆建设顺利推进。高度重视人口和计划生育工作，人口自然增长率控制在4‰以内。公共卫生体系继续完善，突发公共卫生事件预防和处置能力进一步加强，新型农村合作医疗试点工作顺利推进。体育事业健康发展，成功举办了全国游泳冠军赛暨奥运选拔赛、晋城市第三届全民运动会等体育活动。严格责任制落实和责任追究，安全生产保持了连续四个平稳年。双拥’和‘双服务’活动成效显著，国防教育和民兵预备役建设进一步加强。广播电视、民族宗教、妇女儿童、档案史志、外事侨务、文物保护、人民防空、气象、统计、地震、老龄、残疾人等各项工作都取得了新的成绩。

　　──人民生活水平稳步提高。坚持把就业作为发展的重要目标，五年累计新增就业岗位13.6万人，城镇登记失业率稳定在2％以内。养老、失业、医疗、工伤保险的制度框架和管理体系初步建立，保障覆盖面逐年扩大，保障能力有效增强，社会保险费累计结存达到8.6亿元，是“九五”末的11倍。城乡低保稳步推进，2005年城市和农村受保障人数分别达到3.1万人和4.3万人。社会救助体系进一步完善，帮贫济困活动深入开展。城镇居民人均可支配收入由2000年的4842元增加到2005年的8911元，年均递增13％；农民人均纯收入由2000年的2629元增加到2005年的3593元，年均递增6.4％。“十五”末累计建成宽裕型小康乡镇20个，宽裕型小康村535个，有4.8万人实现了稳定脱贫。城、乡居民人均居住面积分别达到28.3平方米和31.2平方米，高于全省平均水平。居民通信、交通和服务消费大幅度增加。社会消费品零售总额由2000年的43.4亿元增加到2005年的75.1亿元，年均递增11.6％。城、乡居民恩格尔系数2005年分别为33.3%和35.7%，比2000年分别下降1个和4.1个百分点。

　　──民主法制和精神文明建设不断加强。市政府主动向市人大及其常委会报告工作，自觉接受人大的监督。积极支持人民政协的工作，加强了同各民主党派、无党派人士和群众团体的联系，注重听取各方面的意见和建议。五年来，共办理人大代表建议1035件，政协委员提案1155件。积极推进基层民主政治建设，政务公开、厂务公开和村务公开不断完善。“四五”普法成效显著，全民法制意识进一步增强。政府决策继续向科学化、民主化迈进，对涉及群众利益的重大事项实行了公示和听证制度。规范和完善了行政执法责任制，依法行政工作进一步强化。加强社会治安综合治理，严厉打击各类犯罪行为，荣获全国社会治安综合治理工作最高奖──“长安杯”。强化行政监察，重视审计工作，廉政建设和反腐败斗争取得显著成效。以文明城市创建为目标，深入开展文明单位、文明社区和文明村镇创建活动，精神文明建设取得新的成果。

　　各位代表，五年来，我们在国民经济和社会发展上取得了显著成就。可以说，这五年是我市综合实力提升最快、城乡面貌变化最大、人民群众得到实惠最多的时期，不仅使我市的综合实力大大提高，而且使加快发展的基础更加坚实，为我市“十一五”时期加快发展创造了良好的条件。这些成就的取得，是贯彻执行党中央、国务院和省委、省政府一系列方针政策的结果，是中共晋城市委正确领导和市人大、市政协支持监督的结果，是全市人民同心同德、奋力拼搏的结果。在这里，我代表市人民政府，向全市人民，向各民主党派、工商联、各人民团体和社会各界人士，向所有关心和支持晋城发展的同志们、朋友们，表示衷心的感谢！

　　五年的实践，使我们对市情的把握更加准确，对经济发展规律的认识更加深刻。我们深深感到，加快晋城发展步伐，必须解放思想，更新观念，把科学发展观和晋城实际紧密结合起来，不断探索符合晋城实际的经济社会发展道路；必须抢抓机遇，善抓机遇，坚持把发展作为第一要务，用发展的办法解决前进中的问题；必须深化改革，扩大开放，努力消除阻碍发展的体制机制束缚，不断为经济社会发展注入生机活力；必须以人为本，和谐发展，注重解决关系人民群众切身利益的突出问题，处理好人与人、人与社会、人与自然等各方面的关系。

　　同时，我们也清醒地看到，“十五”时期全市经济社会发展中仍然存在着一些突出的困难和问题。集中反映是：结构性矛盾尚未从根本上得到解决，单一产业结构应对经济周期性波动的能力较差；农民增收难度加大，建设社会主义新农村任务艰巨；经济增长方式还比较粗放，资源节约和环境保护的压力较大；改革相对滞后，开放仍然不足；经济与社会发展还不够协调，因利益调整引发的社会矛盾增多。这些困难和问题，有的是长期积累下来的，有的是体制转轨和结构调整过程中难以完全避免的，有的是由于工作中的不足造成的。需要今后采取切实措施，努力加以解决。

　　二、对今年政府工作的建议

　　今年是实施“十一五”规划的第一年，也是我市实现跨越式发展的关键一年，改革发展稳定的任务十分繁重。市委对今年政府工作的基本要求是：以邓小平理论和“三个代表”重要思想为指导，坚持以科学发展观统领经济社会发展全局，坚持以经济建设为中心，坚持抓好发展这个第一要务，进一步解放思想、更新观念，在调整产业结构上下功夫，在社会主义新农村建设上下功夫，在城市建设和管理上下功夫，在深化改革、扩大开放上下功夫，在建设资源节约型和环境友好型社会上下功夫，在构建和谐晋城上下功夫，努力实现经济社会又快又好发展，为“十一五”开好局、起好步。

　　根据这个总体要求，市政府经过认真研究，对今年的政府工作提出以下建议。

　　今年全市经济社会发展的主要预期目标：生产总值增长12%，单位生产总值能耗下降5.8%，单位生产总值污染物排放总量下降10%，财政总收入增长15%，一般预算收入增长14%，工业增加值增长17%，全社会固定资产投资增长25%，社会消费品零售总额增长13%，外贸进出口总额增长13.3%，城镇居民人均可支配收入增长12%，农民人均纯收入增长8%，人口自然增长率3.77‰，市区空气质量二级以上天数达到280天。

　　今年的工作重点要“瞄准一个目标，实施三个战略，主攻双百工程，培育八大产业”。

　　———瞄准一个目标，就是要瞄准建设经济发达、人民富裕、生态良好、社会和谐的新兴现代化城市的奋斗目标。

　　———实施三个战略，就是要认真实施扶优扶强战略、开放带动战略和科教兴市战略。通过实施扶优扶强战略，以园区经济、走廊经济、循环经济和品牌经济为切入点，培育一批大企业、大集团，增强市场竞争力和调控力。壮大县域经济，奠定加快发展的基础；通过实施开放带动战略，创优发展环境，大力招商引资，用别人的钱办我们的事，让投资者发财，带动我们发展，实现互利共赢；通过实施科教兴市战略，用先进适用技术改造提升传统产业，培育发展新兴产业和高新技术产业。抓好各类人才的培养、引进和使用，为经济社会发展提供人才和智力支撑。

　　———主攻双百工程，就是要围绕抓好100个重点煤炭企业和100个重点非煤企业的部署，突出抓招商，重点抓项目，以大上项目和上大项目来支撑产业的崛起，来保障经济的腾飞。煤炭企业要通过资源整合，集团重组，增强发展后劲。非煤企业要通过拓展规模，争创品牌，挖掘发展潜力。

　　———培育八大产业，就是要下大力气培育无烟煤、煤化工、电力、中高档铸造、高新技术、新型建材、农副产品加工和旅游产业。经过几年的努力，把我市建设成为全球最大的以煤为原料的高浓度氮肥基地，全国重要的无烟煤基地、煤化工基地、煤层气基地、火电基地和中高档铸件基地，以及全国有影响的电子产业基地。

　　做好今年的政府工作，要以科学发展观为指导，坚持以下原则：一是要坚持贯彻国家宏观调控政策与从本地实际出发相结合的原则。把国家的宏观调控作为加速本地发展的“推进器”，善于在宏观调控中审时度势，把握好方向，把握好力度，把握好机遇，努力推进全市经济社会又快又好地发展；二是要坚持统筹全局与重点突破相结合的原则。正确处理改革发展稳定的关系，善于抓住抓准事关全局的重大问题求突破，以改革开放为着力点推动跨越式发展，以城乡、区域和经济社会协调发展为切入点让广大群众共享发展成果；三是要坚持立足当前与着眼长远相结合的原则。今年各项工作的安排和实施，都要和“十一五”规划目标相衔接、相协调，积极进取，注重实效，以扎扎实实的年度工作为圆满完成“十一五”规划奠定良好的基础。

　　今年要着力做好以下几个方面的工作。

　　（一）以加快建设煤化工基地为突破口，在调整产业结构上下功夫

　　加快产业结构优化升级，是实现经济增长方式转变、提高城市竞争力的必由之路。今年，要以建设全国重要的煤化工基地为突破口，以“四大基地”、“八大产业”为重点，以“双百工程”为支撑，加快产业结构调整步伐。

　　着力提升煤炭产业集中度和产业素质。切实抓好煤炭资源整合，提高煤炭产业集中度，改善煤矿技术装备水平，增强市场竞争力和调控力。按照省政府的要求，坚决完成淘汰小煤矿和关闭压减矿井任务。加快改造和新建一批大中型矿井，大阳、唐安、伏岩山、苏村等12座续建矿井要建成投产，西城、小西等4座重点新建矿井要按时开工。全市煤炭资源回采率、入洗率和转化率分别达到52%、30%和25%，地方煤矿采掘机械化水平达到30%，建设安全质量标准化矿井100座。

　　积极发展以煤化工为重点的新兴优势产业。把煤化工产业作为重中之重，按照晋城煤化工产业发展规划，以化肥为基础，大力发展醇醚燃料和精细化工，搞好煤层气开发和综合利用。今年，要重点抓好已投产项目的达产达效，抓好天脊晋城化工“1830”联产10万吨甲醇及1.2万吨三聚氰胺、兰花田悦“1830”、兰花科创100万吨二甲醚一期、晋煤集团10万吨煤基合成油等已开工项目的建设，开工建设晋城煤化工“4060”等新建项目，抓紧天脊晋城化工“5080”项目的前期工作。电力产业要加快阳电二期建设进度，加快国投华实晋城热电联供项目、鲁能电厂项目、高都煤矸石电厂项目和阳城煤矸石电厂项目的前期工作，全力争取国家的核准。冶铸产业要开工建设福盛钢厂三期50万吨中宽带轧钢、清慧汽车配件公司6万吨精密汽车配件、泽州世纪管业10万吨水冷离心球墨铸管等一批重点项目。高新技术产业要加快富士康晋城科技工业园建设，确保年内顺利投产。对这个项目，各有关部门要继续特事特办，一路绿灯。新型建材业要重点抓好阳城建瓷工业园区和高平东山、泽州康达和阳城西城等三条新型水泥熟料生产线建设，加快开发新型建筑材料，实现产品结构由单一、低档向多元化、中高档方向转变。农副产品加工业要以丝麻、果品、肉类、中药材、小杂粮为重点，加快绿洲大麻等丝麻纺织企业的改制步伐，壮大厦普赛尔、鸿生淀粉、华萸饮品、塔王醋业等龙头企业，提高市场竞争能力。

　　继续壮大旅游产业。抓住我市获得“中国优秀旅游城市”称号的大好机遇，继续完善城市服务功能，改善城市旅游环境，加强旅游设施建设，巩固和扩大创优成果。重点抓好旅游产品促销工作，主打皇城相府和围棋发源地、王莽岭两大品牌，加快建设以皇城相府为主包括郭峪城、柳氏民居、蟒河、历山、九女湖在内的旅游西线，以珏山、青莲寺为主包括山里泉、炎帝陵、长平古战场、白马寺森林公园在内的旅游中线，以围棋发源地、王莽岭为主包括锡崖沟、凤凰欢乐谷、黄围山在内的旅游东线，尽快形成“西、中、东”三线互动、三线相连的旅游经济圈，力争把皇城相府建成5A级景区，把王莽岭建成4A级景区，塑造“山西风光·美在晋城”的旅游形象。积极与周边地区合作共建无屏障旅游区，推动旅游产业快速发展，真正把旅游资源优势转化为产业优势和经济优势。

　　加快发展商贸流通业和现代服务业。加大运用新型商业业态、现代经营方式和服务技术改造商贸流通业力度，开工建设晋城豪德光彩贸易广场，打造现代物流园区和物流中心。推进城市商业网点建设，积极发展专业连锁超市，规范发展小商品市场，优化社区商业网点布局。以实施国家“万村千乡”市场工程为契机，积极推动城市商贸流通网络向农村延伸，建设以县城店为龙头、乡镇店为骨干、村级店为基础，集中采购、统一配送的农村商业流通网络。积极发展房地产、金融、证券、中介、信息咨询、文化娱乐、社区服务等现代服务业，努力提高第三产业占生产总值的比重。

　　加强基础设施建设。今年，要集中力量抓好关系全局的12项重点工程。其中，续建5项，分别为张峰水库、阳电二期、晋济高速公路、城市污水处理厂和城市生活垃圾无害化处理工程；新开工7项，分别是富士康晋城科技工业园、兰花集团二甲醚项目、晋城豪德光彩贸易广场、晋城矿区采煤沉陷区综合治理工程、阳城至侯马高速公路、端氏220千伏输变电工程和晋城二中综合教学楼。

　　（二）以增加农民收入为突破口，在社会主义新农村建设上下功夫

　　建设社会主义新农村是全面建设小康社会的重大历史任务。今年，要按照“生产发展、生活宽裕、乡风文明、村容整洁、管理民主”的要求，贯彻工业反哺农业、城市支持农村的方针，加大对“三农”的支持力度，推进农村制度创新，确保我市社会主义新农村建设工作起好步。

　　扎实做好社会主义新农村建设起步工作。认真做好社会主义新农村建设规划，年内完成25个乡镇、200个村的规划编制，乡镇和村的规划率分别达到75%和27%。在建设重点上，完成“三个100”任务，即选择100个发展较好的村进行新农村试点示范，建成100个新农村达标村，完成100个缺乏生存条件的自然村搬迁。突出村容村貌整治，完善农村生产生活设施，使新农村全部实现“六通”、基本具备“六有”、初步达到“六化”。即通水、通电、通路、通电话、通班车、通信息；有幼儿园、有敬老院、有卫生所、有文化大院、有健身场所、有洗浴美发室；住房舒适化、饮水安全化、能源新型化、街道明亮化、环境整洁化、村庄园林化。

　　加快发展现代农业。加大对农业的扶持力度，继续落实对农民种粮、购买农机具和发展畜牧业的补贴政策。加快农业结构调整步伐，充分发挥比较优势，抓好畜牧、蚕桑、蔬菜、小杂粮、干鲜果、中药材6大特色基地建设，尤其是要继续大力发展畜牧业，使其在农业中的比重进一步提高。推进农业产业化经营，培育壮大一批农业产业化龙头企业，发展一批农民专业合作经济组织，提高农民走向市场的组织化程度。加强农业科技引进和推广工作，实施好农业科技入户工程和农民素质提高工程。大力发展农村二、三产业，壮大县域经济，推进农村劳动力向非农产业和城镇有序转移，拓宽农民增收渠道。

　　加强农业和农村基础设施建设。抓好张峰水库、磨滩水库和东焦河水库建设。启动100万亩耕地综合生产能力建设工程，新发展农业节水面积3万亩。实行最严格的耕地保护制度，新增土地复垦面积1万亩。以4个县城和巴公、南村、北留、嘉丰、马村、河西等重点小城镇为载体，完善基础设施和公共服务设施，强化辐射带动作用。积极发展农村教育事业，切实解决贫困家庭子女入学难问题。加强农村公共卫生体系和基本医疗服务体系建设，改善农村卫生条件。完成村村通水泥路或油路的后续扫尾工作，加快公路客运场站建设，实现“路通车通”。启动农村饮水安全工程。新建5000个户用沼气池，积极发展规模化沼气工程。

　　深化农村综合改革。坚持和完善农村土地承包制度，依法规范土地承包经营权流转程序和行为，改革和完善征地补偿制度，切实维护农民合法权益。完善农民负担监督管理机制，规范涉农税费收缴行为。抓好乡镇机构、县乡财政管理体制和农村义务教育等改革试点工作，巩固农村税费改革成果。

　　（三）以改善人居环境为突破口，在城市建设和管理上下功夫

　　城市化是工业化、现代化的重要标志。今年，要以扩容提质为核心，继续强化中心城市建设，完善城市功能，塑造城市特色，提升城市品位，改善人居环境，充分发挥中心城市在城镇化进程中的带动作用。

　　切实搞好城市规划工作。按照高起点、大手笔、精品化的思路，修编市域城镇体系规划，完成城市近期建设控制性详细规划，做好重点地段城市设计、“城中村”改造规划、城市色彩规划、城市户外广告规划等专项规划。加强城市规划的实施管理，推行城市规划公示制度、建设项目选址制度、城市规划年度实施计划制度和建设项目竣工规划认可制度，维护城市规划的权威性和严肃性。

　　进一步加大经营城市力度。管理和配置好城市资源，用活包括城市土地在内的所有资产，加大土地收购储备力度，严格执行经营性用地“招、拍、挂”制度，不断提升城市资源价值。今后，凡城市主、次干道临街用地一般不再用于多层住宅建设，以提高临街用地的经济效益，改善城市街道的景观效果。公开拍卖户外广告位经营权和城市道路、桥梁、广场、游园冠名权，提高城市基础设施自我积累、自我发展的能力。全面开放城市建设投资领域，推进市政公用事业特许经营和市场化改革，力争用3年左右的时间建立完善市政公用事业的市场竞争机制、企业经营机制和政府监管机制。今年，市政公用事业单位改革试点要迈出实质性步伐。

　　精心实施城市建设重点工程。一是“蓝天碧水工程”。通过关停搬迁市区工业污染源、扩大集中供热覆盖面、加快生活垃圾无害化处理、控制扬尘污染、加大机动车尾气治理、鼓励汽车使用煤层气等措施，确保市区空气质量二级以上天数达到280天以上，空气综合污染指数下降到2.8以下。巩固市区‘两河’治理成果，确保水域面积稳定和水质达标。加强城市饮用水源地保护，确保市区饮用水水质达标率100%；二是“畅通工程”。今年要新建白水街，改造景西路，延伸前进路，建设开发区的红星东街东延伸段和新市东街东延伸段，新增城市道路4.3公里。同时，要通过控制交通流量、加强停车场（点）建设和管理、优先发展公共交通等措施，有效缓解“瓶颈”路段高峰期交通拥堵问题；三是“绿化工程”。加快建设泽州公园、儿童公园、流碑亭公园等绿化精品，建成5个绿化达标小区和5个绿化达标庭院，新增绿地面积330公顷。拆除泽州公园和植物园围墙，建设开放性公园。开展市树、市花评选活动，提升城市品位。在城市绿化中，要少种草、多栽树、栽大树，提高生态效益；四是“净化工程”。加快垃圾处理厂建设进度，垃圾中转站尽快全部投入运营，垃圾填埋区9月份实现无害化处理。市区煤气补充气源续建工程要抓紧建成供气。加快市区煤层气利用项目建设步伐，年内完成中压管网铺设和30%的庭院管网铺设，具备供气条件。

　　不断提高城市管理水平。坚持建管并重，标本兼治，构建城市管理长效机制。加大市容环境整治力度，重点整治市区乱停乱放、乱搭乱建、乱贴乱画、乱摆摊点、占道经营、无证经营等群众反映强烈的问题。城市管理要向背街小巷延伸，消除管理盲区和盲点。推进城市社区建设，夯实城市管理基础。深入开展“做文明晋城人，建文明晋城市”活动，引导广大市民增强城市意识，提高文明素质，营造热爱晋城、关心晋城、建设晋城的社会氛围，全方位提高城市文明程度，推进文明城市创建工作。

　　（四）以进一步解放思想、更新观念为突破口，在深化改革、扩大开放上下功夫

　　深化改革、扩大开放是增强经济社会发展活力的源泉所在。按照省委、省政府把晋城建设成为山西改革开放桥头堡的要求，今年，要采取有效措施，大力推进改革，扩大对外开放，用改革释放和激活发展的潜能和动力，用开放推动和促进发展的速度和进程。

　　加大对外开放力度。认真贯彻落实全省对外开放工作会议精神，充分认识扩大开放对实现我市经济社会又快又好发展的战略意义和关键作用，切实把扩大对外开放作为推进全市科学发展的重大举措来抓。大力倡导“服务是政府的主要产品”、“服务是第一投资环境”等理念，规范行政行为，提高行政效率，优化投资环境，营造“重商、亲商、安商、富商”的良好氛围。进一步完善重点产业发展规划，强化开发区和工业园区基础设施，做好项目前期准备及项目用地的置换和储备，为招商引资提供载体。扎实做好招商引资项目的开发、设计、筛选和发布工作，舍得拿出优势项目和优质资产招商引资，吸引更多的大企业、大集团落户晋城，引进战略投资者，以存量带动增量。创新招商引资方式，多管齐下，主动出击，继续组团参加厦洽会、高交会、乌洽会等有影响的大型招商贸易洽谈活动。特别是要精心准备，积极参加省政府今年在上海、香港举办的招商活动，务求取得实效。继续优化出口产品结构，扩大铸管、轻纺产品和汽车、空调压缩机零配件的出口规模，培育农副产品和高新技术产品出口基地。立足我市的产业特色和区位优势，实施好“南下战略”，力争在接轨中原城市群、融入中原经济圈方面有实质性进展。

　　加快推进企业改革。今年是全市的企业改革攻坚年，按照改革的总体部署，国有和城镇集体企业要有步骤地完成企业产权和职工身份双置换工作，进行彻底的产权制度改革。市属国有企业要率先做好改革工作。兰花科创要推进和完善股权分置改革。乡村集体企业要有70％以上完成改制改组任务，切实增强发展活力。煤炭企业要按照“资源有偿化，企业股份化，区域集团化”的思路，在完成资源整合和有偿使用工作的基础上，同步推进产权制度改革。积极推进大企业、大集团战略，特别是太行无烟煤集团要完善法人治理结构，抓好规范运行，在推动煤炭资源整合、煤炭产业升级和煤化工产业发展方面发挥主力军作用。

　　大力发展非公有制经济。充分认识非公有制经济在促进经济增长、扩大就业、繁荣市场等方面的重要作用，进一步放宽市场准入，加大扶持力度，营造有利于非公有制经济加快发展的宽松环境，鼓励非公有制经济参与国有和城镇集体企业产权制度改革，参与煤炭资源整合和有偿使用，参与“四大基地”、“八大产业”建设，参与城市基础设施和市政公用设施建设，参与农业综合开发，逐步提高非公有制经济所占比重。

　　深化投融资体制、财政体制和国有资产监管体制改革。规范政府投资行为，强化企业投资主体地位。市、县两级政府要建立完善银企联席会议制度，搭建重点建设工程、重大产业项目和中小企业融资平台。驻市的国有商业银行、股份制银行和政策性银行，既要有效地防范金融风险，又不能为追求低风险而不为中小企业放贷，必须采取切实有效的措施，加大对地方经济的支持力度。城市商业银行和农村信用合作社要继续深化改革，发挥政策灵活、贴近基层的优势，为中小企业发展和新农村建设做出积极贡献。全面开放投资领域，进一步畅通社会投资渠道，特别是要鼓励引导民间资本更多地投向生产领域。进一步探索完善财权与事权相匹配、经济发展与财力增加相衔接的财政管理体制。启动陵川县县乡财政管理体制改革试点。完善公共财政框架，优化财政支出结构。进一步深化部门预算、国库集中支付、政府采购和收支两条线改革。切实加强财政投资项目评审和支出绩效评价工作，提高资金使用效益。进一步完善国有资产监管体制，建立健全国有资本经营预算制度、经营业绩考核体系和国有资产重大损失责任追究制度。

　　（五）以转变经济增长方式为突破口，在建设资源节约型和环境友好型社会上下功夫

　　建设资源节约型和环境友好型社会是落实科学发展观的基本要求。要突出工作重点，狠抓措施落实，真正把经济增长方式转移到集约增长的轨道上来。

　　大力发展循环经济和清洁生产。制定出台节约资源能源、推进循环生产的实施意见，积极在企业层面推行清洁生产，在产业层面构建生态产业链条，在社会层面建设循环经济。重点在煤炭、电力、冶铸、建材、丝麻纺织等行业，按照循环经济的要求，改进设备、工艺技术和生产流程，培育一批循环经济示范企业。抓好煤炭等资源型初级产品的加工转化，抓好煤矿废水利用、瓦斯综合利用、焦炉尾气利用、粉煤灰利用和煤矸石利用等资源综合利用技术的推广应用。今后，对新建的工业园区、新上的工业项目都必须严格要求，凡不符合循环经济和清洁生产要求的企业与项目，一律不准开工。

　　加快节约型社会建设步伐。突出抓好煤炭、冶铸、焦化、化工、电力、建材等重点行业的节能、节材工作，对重点单位制定科学的耗能指标，建立健全计量管理制度、能源消费统计制度和节能工作奖罚制度。鼓励发展节能型交通运输工具，积极推广商业、民用节能技术。大力推进城市节水工作，加大关闭自备水源井力度，推广节水型器具，推进中水循环利用。发展节能、省地型住宅和公共建筑，推进建筑节材。积极发展农业节水灌溉。加强土地资源管理，促进城乡建设用地、交通等基础设施用地和工矿企业用地的集约利用。深化资源价格管理，促进资源节约工作。为保护煤炭资源，稳定煤炭价格，今后只考虑给煤化工、煤电一体等新建煤炭加工转化项目配置适量的煤炭资源和产能。凡单纯开采煤炭、不同步进行加工转化的项目，一律不批，一律不上。

　　加强生态建设和环境治理。按照省政府把晋城建设成为绿色山西示范区的要求，全面实施林业“六大工程”，推动山上治本，加快身边增绿，特别要在经济比较发达、人口相对集中的地方多栽树，栽大树，一次成林，一次成景。全年完成造林10万亩，四旁植树1000万株，建成30个新农村生态园林示范村。加快矿区生态环境治理，促进生态恢复。严格控制中心城市废水、废气和固体污染物的排放，中心城市建成区工业污染企业要有计划地实施关停或搬迁，建成区烟尘控制区覆盖率要达到90%以上。积极实施“碧水工程”。巴公工业园区废水处理厂要保证正常运行，晋煤集团和高平市生活污水处理厂年内要投入使用。启动丹河植物净化工程，力争使丹河水质由5类恢复到4类。加强沁河流域环境监管，所有新建项目必须严格进行环境影响评价，确保沁河水质稳定在3类。抓好生态示范区建设及旅游沿线生态环境保护工作，陵川、沁水两个国家级生态示范区今年要通过国家验收。

　　（六）以推进各项社会事业发展为突破口，在构建和谐晋城上下功夫

　　在加快经济发展的同时，要坚持以人为本，注重发展社会事业，高度重视解决涉及人民群众切身利益的热点问题，维护社会稳定，构建和谐晋城。

　　大力实施科教兴市和人才强市战略。深化市直科研机构改革，有效整合科技资源。广泛建立企业与高校、科研机构及企业间的技术联盟，发挥企业在技术创新中的主力军作用。实施一批各级各类科技项目，提高产品科技含量，增强产业竞争能力。通过合作攻关和引进吸收，尽快掌握煤化工、煤基合成油、煤层气开发等方面的关键技术，推进产品升级和产业延伸。牢固树立人才资源是第一资源和“不求所有，但求所用”的观念，紧紧抓住培养、引进、使用三个环节，加强人才队伍建设，为经济社会发展提供人才支撑。

　　优先发展教育事业。以全面提升义务教育、加速普及高中阶段教育、大力发展职业教育和积极创办高等教育为重点，促进教育事业加快发展。抓好晋城二中、中原街中学、凤台中学、星河学校等10所中小学校新建扩建项目，缓解市区中小学的班容量过大问题。继续调整农村中小学布局，建设100所标准化寄宿制学校，改造农村中小学危房7.4万平方米。继续大力推进民办教育，增强教育事业发展活力。积极发展职业教育和成人教育，为经济社会发展培养实用型、应用型人才。积极筹建本科大学，改善我市教育结构，提升我市文化品位。

　　加快发展文化、卫生、体育等社会事业。抓好理想信念和社会主义荣辱观教育，深入开展群众性精神文明创建活动。推进文化体制改革，促进文化事业和文化产业协调发展。组织好人民作家赵树理诞辰100周年纪念活动，承办好山西省第五届广场文化艺术节。加强农村卫生工作和城市社区卫生工作，完善疾病预防控制体系、卫生执法监督体系和突发公共卫生事件应急体系，提高卫生保障水平。完成市传染病医院建设。总结泽州和城区试点经验，在高平、阳城和沁水开展新型农村合作医疗工作，新增受惠人口81万。高度重视人口和计划生育工作，稳定低生育水平，提高出生人口质量。大力开展全民健身活动，积极备战山西省第十二届运动会，办好全市第三届农民运动会。重视国防教育，完善国防动员机制，深入开展“双拥”和“双服务”活动，加强人防工作。继续做好广播电视、民族宗教、妇女儿童、档案史志、外事侨务、文物保护、气象、统计、地震、老龄、残疾人等各项工作。

　　千方百计扩大就业。继续实施积极的就业政策，认真落实财政投入、小额贷款、免费培训和税费减免等优惠政策，完善公共就业服务体系，积极开展就业再就业培训，充分发挥劳动力市场等中介机构的作用，注重发展就业容量大的劳动密集型产业、服务业和中小企业，改善就业环境，拓宽就业渠道。注重解决体制转轨遗留的下岗失业人员再就业问题和关闭破产、重组改制企业的职工安置问题，高度重视高校毕业生、退役军人、城镇新增劳动力和进城农民工的就业指导与服务。今年，要新增城镇就业岗位2万个，转移就业和对外劳务输出5万人，城镇登记失业率控制在4％以内。

　　进一步完善社会保障体系。全面推进养老、失业、工伤、医疗和生育保险制度建设，切实保证各项社会保险基金的足额征缴和及时支付。继续提高各类所有制企业的参保率，扩大社会保险覆盖面。完成下岗职工基本生活保障制度向失业保险制度并轨的扫尾工作，维护职工利益。继续巩固和完善城乡最低生活保障制度，高度重视城镇化进程中失地农民的保障问题。关注弱势群体，完善农村五保供养、城乡医疗救助、灾民救济等救助制度，落实军烈属优抚政策，发展社会福利事业和慈善事业，开展多种形式的捐助和帮扶活动。

　　切实抓好安全生产。安全生产只有起点没有终点，安全这根弦永远不能有丝毫的松懈。要进一步加大安全生产投入力度，完善安全生产监管体系，落实安全生产责任制，严格安全生产责任追究，努力实现第五个安全生产平稳年。特别是要切实强化煤矿安全工作，加大安全质量标准化矿井建设力度，加强高瓦斯、高突出、高危险矿井的管理，严厉打击非法生产，坚决控制超能力生产。兰花集团由南非引进的瓦斯隔爆技术，示范成功后要在全市推广。高度重视道路交通安全工作，重点抓好宣传教育和危险路段整治，严防重特大事故的发生。同时，下大力抓好特种设备、烟花爆竹、危险化学品、非煤矿山、建筑施工、消防安全和公众聚集场所等专项安全整治。通过采取切实措施，确保省控安全生产目标的圆满完成。

　　全力维护社会稳定。重视矛盾纠纷排查调处工作，正确处理新时期社会矛盾，依法解决好农村土地征用、城镇房屋拆迁、国有企业改制、乡村集体煤矿承包等涉及群众切身利益的纠纷和问题。认真落实信访工作责任制，规范信访秩序，做好信访工作，加大对不理民事者的责任追究力度。完善社会稳定预警体系，提升应急处理能力。进一步强化社会治安综合治理，加快建设社会治安防控体系,深入开展“严打”整治专项斗争，依法严厉打击严重暴力性犯罪、经济犯罪和盗窃、抢劫等多发性侵财犯罪，深入开展“扫黄打非”和禁毒斗争。

　　加强民主法制建设。政府要主动向人大及其常委会报告工作，自觉接受人大常委会和人大代表的监督。充分发挥人民政协、各民主党派、人民团体在政治协商、参政议政和民主监督中的积极作用。继续加强基层民主，完善基层群众性自治组织和企事业单位民主管理制度，完善政务、村务、厂务公开和基层民主选举制度。进一步加强政府法制建设，推进依法行政，规范行政行为。搞好“五五”普法教育，提高全民法律素质。促进司法公正，维护司法权威。

　　在促进全市经济社会全面发展的同时，要围绕人民群众最关心、最直接、最现实的利益问题，认真办好20件实事：一是农民技术培训10万人，引导性培训3万人，转移农村富余劳动力1万人；二是解决10万农村人口饮水困难和饮水安全问题；三是发展农村连锁便民店258个，健全农村消费品流通网络；四是新增高平、阳城、沁水3个县（市）开展新型农村合作医疗，参合率达到80％以上；五是1万农村贫困人口实现稳定脱贫，整体搬迁100个自然村；六是对农村4.5万名特困群众实行低保，对4777名“五保”对象实行应保尽保；七是新建改建城镇中小学标准操场53个；八是继续扶持规模养殖和园区养殖，确保农民人均畜牧业纯收入比上年增长9％；九是新建畜牧兽医中心站16个，完善畜牧防疫服务体系；十是新建5000个户用沼气池，改善农村能源结构；一是大力实施‘蓝天碧水工程’”，市区空气质量二级以上天数达到280天以上，空气综合污染指数下降到2.8以下，市区饮用水水质达标率100%；十二是加快建设城市煤气补充气源工程；十三是完成白水街、景西路、前进路、新市街、红星街等5段道路建设；十四是实施市区绿化工程，新增绿地330公顷；十五是加快建设城市生活垃圾无害化处理工程；十六是实施工矿企业棚户区改造工程；十七是建设安康居住工程住房37.6万平方米，解决4584户居民住房困难；十八是实施晋城二中、中原街中学、凤台中学等学校的改建、扩建工程；十九是完成10处公厕和垃圾中转站建设任务；二十是提高企业离退休人员、城市“低保”对象、军队移交政府安置的离退休人员、残疾军人等各类优抚对象的生活补助标准。

三、关于《晋城市国民经济和社会发展第十一个五年规划纲要（草案）》的说明

　　中共晋城市委四届六次全会通过的《中共晋城市委关于制定国民经济和社会发展第十一个五年规划的建议》，提出了今后五年我市国民经济和社会发展的指导思想、奋斗目标和主要任务。根据《建议》精神，市政府制定了《晋城市国民经济和社会发展第十一个五年规划纲要（草案）》，已提请大会审查。下面，我就几个问题作简要说明。

　　（一）“十一五”规划《纲要（草案）》的编制过程和主要特点

　　市委、市政府对制定“十一五”规划工作高度重视。2005年初，全省“十一五”规划编制工作会议召开之后，市政府就成立了编制“十一五”规划领导组和办公室，组织有关部门对关系全市经济社会发展全局的产业结构调整、煤化工基地建设、和谐社会构建等重大课题进行了前期调研，筛选汇总了“十一五”时期重大建设项目，初步测算了“十一五”时期经济社会发展的指标体系，编制完成了农业、工业、科技、教育等32个行业和部门的专项规划。这些前期工作的完成，为编制《纲要（草案）》奠定了良好的基础。市委四届六次全会后，市政府根据市委《建议》精神，深入研究和编制“十一五”规划，并广泛征求了各县（市、区）、市直各部门、各民主党派、无党派人士和有关专家的意见。《纲要（草案）》的编制过程，是解放思想、发扬民主、集思广益、科学决策的过程。

　　《纲要（草案）》力求反映社会主义市场经济发展和改革开放新形势的要求，体现了宏观性、战略性和政策性，并明确了政府的工作重点和责任。《纲要（草案）》从内容到形式都有所创新。在规划内容上，突出了落实科学发展观和构建社会主义和谐社会的战略要求，突出了加强经济社会发展的薄弱环节，突出了解决人民群众关注的切身利益问题，突出了运用市场机制和注重宏观调控的有机结合。在规划指标上，采用了新的地区经济社会发展指标体系。其中，预期性指标是政府期望的发展目标，主要通过引导市场主体行为来实现。约束性指标是在预期性基础上，进一步明确并强化了政府责任而必须实现的发展目标，主要通过依法加强管理和提供服务来实现。在规划形式上，《纲要（草案）》采取正文加专栏的形式，在专栏中列举了有关发展指标和重点项目，使规划内容更加直观、清晰。同时，《纲要（草案）》的制定以第一次全国经济普查数据为基础，使“十一五”规划更符合我市实际情况，更具有科学性。

　　（二）“十一五”时期经济社会发展的指导原则和主要目标

　　“十一五”时期是我市实现经济社会发展历史性跨越的关键时期。综合分析各种因素，我们既面临许多发展机遇和有利条件，也面临不少制约因素和不利条件，必须保持清醒头脑，进一步增强加快发展的紧迫感、危机感和责任感，抓住机遇，趋利避害，努力开创全市经济社会发展的新局面。

　　《纲要（草案）》全面贯彻落实科学发展观，体现了市委《建议》提出的重要原则，这就是：坚持把科学发展观统领下的争先发展作为鲜明主题，坚持把调整经济结构和转变经济增长方式作为突出主线，坚持把改革开放和科技进步作为主要动力，坚持把统筹城市与农村、经济与社会协调发展作为重大任务，坚持把不断提高人民生活水平作为根本目的。

　　根据市委《建议》确定的指导思想和原则，《纲要（草案）》提出了“十一五”时期我市经济社会发展的主要目标。这些目标主要由经济增长、社会发展、科技进步、资源环境和人民生活5个方面44项指标组成，既与全面建设小康社会的目标相衔接，又反映了经济社会发展的阶段性特征和客观要求。这里主要就两个重要目标作简要说明：

　　一是关于经济增长速度问题。《纲要（草案）》提出， “十一五”时期全市生产总值年均增长12%以上，比全省10%的预期速度高出2个百分点，人均生产总值保持全省领先水平。这个目标是在充分考虑我市发展趋势和各方面条件的基础上，经过反复测算和研究讨论确定的。确定这样的发展速度，既体现了省委、省政府对晋城的殷切期望和全市人民群众的迫切要求，又是我市抓住机遇，争先发展，不断提高城市综合实力和竞争力的需要，同时考虑了实际可能，是积极稳妥的，符合我市加快发展的要求。

　　二是关于节能和环保问题。《纲要（草案）》提出了“十一五”时期全市单位生产总值能耗降低25%、主要污染物排放量减少40%等节能和环保目标。这是根据中央、省委的战略要求和我市资源环境约束日益加重的具体实际提出的，体现了建设资源节约型、环境友好型社会和实现可持续发展的需要。尽管实现这一目标的难度不小，但我们有信心、有决心努力完成。

　　（三）“十一五”时期的战略重点和主要任务

　　《纲要（草案）》对“十一五”时期的经济建设、社会发展、改革开放等方面，都作出了全面部署，提出了明确的任务和措施。

　　一是建设社会主义新农村。《纲要（草案）》把解决“三农”问题放在各项战略任务的首位。强调坚持城乡统筹发展，按照“二十字”方针的要求，因地制宜，分类指导，加快社会主义新农村建设步伐。要继续推进农业结构调整，大力发展农业产业化经营，加快农业科技进步，积极发展现代农业。要充分挖掘农业内部增收潜力，积极发展农村二、三产业，加大扶贫开发力度，增加农民收入。要加强农业和农村基础设施建设，加快发展农村教育、卫生、文化、体育和社会保障等公共事业，改善农村生产生活条件。要全面推进农村综合改革，基本完成乡镇机构、农村义务教育和县乡财政管理体制等改革任务。公共财政要更多地向“三农”倾斜，公共服务要更大范围地覆盖农村，全社会要更加有力地支持农村发展。

　　二是加快经济结构调整和经济增长方式转变。针对我市经济发展中存在的结构不合理和增长方式粗放问题，《纲要（草案）》提出，在“十一五”期间，要按照走新型工业化道路的要求，重点发展“四大基地”、“八大产业”，积极实施“双百工程”，从优化产业结构中求发展，从节约资源、保护环境中求发展。《纲要（草案）》对做大做强优势产业、改造提升传统产业、扶持培育高新技术产业、加快发展服务业、着力优化产业组织结构等方面的主要任务和建设布局作了部署，规划了一批重大建设项目。《纲要（草案）》把建设资源节约型、环境友好型社会摆在突出位置，提出了重点行业的节能要求，规划了一批循环经济示范试点项目和生态建设、环境保护重点工程。认真落实这些任务和措施，一定会有效地提高资源利用效率，一定会有效地改善生态环境，为子孙后代留下蓝天绿地、碧水青山。

　　三是大力推进城镇化进程。加快城镇化进程，是关系改革开放和现代化建设全局的重大问题。《纲要（草案）》提出要按照科学规划、合理布局、设施配套、环境优美、增强特色的原则，加快推进城镇化进程。《纲要（草案）》对提高中心城市综合竞争力、搞好县城和中心集镇建设都提出了明确的要求。特别提出要优先发展中心城市，构建组团式的城市格局。《纲要（草案）》还对构建城市产业集群、大力发展县域经济作了全面部署，强调以工业化支撑城镇化，以城镇化承载工业化，双轮驱动，相辅相成，协调发展。

　　四是深化改革和扩大开放。改革开放相对不足是制约我市经济社会发展的重要因素之一。《纲要（草案）》对进一步深化改革、扩大开放提出了明确的要求。要深化国有集体企业、投融资体制、财政金融体制等重点领域和关键环节的改革，大力发展非公有制经济，加快形成有利于转变经济增长方式、促进全面协调可持续发展的体制机制。特别强调煤炭产业要按照“资源有偿化，企业股份化，区域集团化”的思路，把资源整合、有偿使用、产权改革和组建集团结合起来，实现投资主体多元化，增强主导产业市场竞争力和调控力。要进一步强化开放意识，实施互利共赢的开放战略，创新招商引资方式，推进开发区和工业园区建设，提高招商引资质量，扩大进出口贸易，加强与国内外的经济技术合作，积极实施“南下战略”，以开放促改革，以开放促发展。

　　五是努力构建和谐晋城。这是推进经济社会发展的重要目标和保障。《纲要（草案）》提出，要千方百计扩大就业，完善社会保障体系，大力发展教育、文化、卫生等社会事业，加强公共安全管理，加强社会主义民主政治建设，完善社会管理体制。经过今后五年的努力，我们的社会会更加和谐，全市人民的生活会更加美好。

　　各位代表，做好今年和“十一五”时期的各项工作，必须进一步加强政府自身建设。一是要进一步解放思想，更新观念。重点在“四破四立”上狠下功夫，即坚决破除小进即满、小富则安的思想，牢固树立强烈的发展意识，善于在大发展、快发展中建功立业；坚决破除因循守旧、墨守成规的思想，牢固树立强烈的创新意识，善于从实际出发创造性地开展工作；坚决破除妄自菲薄、无所作为的思想，牢固树立强烈的机遇意识，善于变“看似不能”为“实际可行”；坚决破除“肥水不流外人田”的思想，牢固树立强烈的开放意识，善于在合作共赢中加快发展。通过解放思想，转变观念，在全市营造一门心思抓发展、全力以赴搞建设的良好氛围。二是要进一步转变职能，提高效率。加快推进行政管理体制改革，继续减少和规范行政许可和行政审批，坚决把政府不该管、也管不好的事交给市场、企业、社会组织和中介机构，切实把政府职能转变到经济调节、市场监管、社会管理和公共服务上来。大力推进政务公开，完善政府新闻发布制度和信息公布制度，发展电子政务，提高工作透明度和办事效率。建立健全行政问责制，提高政府执行力和公信力。三是要进一步改进作风，求真务实。各级政府工作人员特别是领导干部要忠于职守，勤勉尽职，全心全意为人民服务。要大力弘扬实干精神，切实精简会议和文件，改进会风和文风，用更多的时间深入基层，深入群众，指导工作，解决问题。要加强部门之间的协调沟通，严肃政务纪律，努力形成政令畅通、协同推进的工作格局。要强化目标管理、效能监察和责任追究，确保目标分解到位、压力传递到位、工作落实到位。四是要进一步从严治政，清正廉洁。建立健全教育、制度、监督并重的惩治和预防腐败体系，认真落实惩治、预防腐败的各项任务和措施。强化行政监察和审计监督，加强政风行风建设，抓好政风行风评议，优化政务环境。今年，要着力解决教育乱收费、医疗高收费等损害群众利益的突出问题，集中开展工程建设、土地出让、产权交易、医药购销和政府采购等领域的治理商业贿赂专项工作。

　　各位代表，回顾“十五”，成就令人鼓舞；展望“十一五”，蓝图催人奋进。我们已经站在新的历史起点，迈上新的辉煌征程。我们坚信，全市人民高举邓小平理论和“三个代表”重要思想伟大旗帜，在中共晋城市委的正确领导下，全面贯彻和落实科学发展观，同心同德，奋发图强，一定能够把“十一五”规划的宏伟蓝图变为美好现实，谱写出建设经济发达、人民富裕、生态良好、社会和谐的新兴现代化城市的新篇章！
